# SisterTalk: final results of a culturally tailored cable television delivered weight control program for Black women

**DOI:** 10.1186/1479-5868-10-141

**Published:** 2013-12-27

**Authors:** Patricia Markham Risica, Kim M Gans, Shiriki Kumanyika, Usree Kirtania, Thomas M Lasater

**Affiliations:** 1 Institute for Community Health Promotion, Brown University School of Public Health, Providence, Rhode Island 02912, USA; 2 The Perelman School of Medicine, University of Pennsylvania, CCEB, 8 Blockley Hall, 423 Guardian Drive, Philadelphia, PA 19104, USA

**Keywords:** Obesity, Overweight, Weight control, Black, African American, Women, Intervention, Randomized controlled trial, Cable TV

## Abstract

**Background:**

Obesity among Black women continues to exceed that of other women. Most weight loss programs created without reference to specific cultural contexts are less effective for Black than White women. Weight control approaches accessible to Black women and adapted to relevant cultural contexts are important for addressing this problem. This paper reports the final results of SisterTalk, the randomized controlled trial of a cable TV weight control program oriented toward Black women.

**Methods:**

A five group design included a comparison group and a 2 × 2 factorial comparison of a) interactive vs. passive programming and b) telephone social support vs no telephone support, with 12 weekly initial cable TV programs followed by 4 monthly booster videos. At baseline, 3, 8, and 12 months post randomization, telephone and in person surveys were administered on diet, physical activity, and physical measurements of height and weight were taken to calculate body mass index (BMI). Analysis of variance (ANOVA) was used to examine differences over time, and between treatment and comparison groups. Dose variables reflecting use of the TV/video and written materials were also assessed.

**Results:**

At 3 months, BMI, weight, and dietary fat were significantly lower and physical activity significantly higher among women exposed to the Cable TV intervention compared to the wait-list comparison group. Significant dietary fat differences were still observed at 8 and 12 month evaluations, but not BMI or physical activity differences. Main effects were not observed for interactive programming or enhanced social support at any time point. Within the intervention group, higher watching of the TV series and higher reading of educational materials were both (separately) associated with significantly lower dietary fat.

**Conclusions:**

Cable TV was an effective delivery channel to assist Black women with weight control, increasing physical activity and decreasing dietary fat during an initial intervention period, but only dietary changes persisted Enhanced social support and the ability to interact with others during the show were not effective complementary intervention components as conducted in this trial. Future research to strengthen the ability of this approach to achieve long term effects may offer even more promising outcomes.

## Background

In the US, the prevalence of obesity has increased dramatically over the past 30 years [1]. While the increasing prevalence of obesity has been observed among all race/ethnic groups, Black women have a higher prevalence of excess weight (78.2% with BMI >25 kg/m^2^) than white women (61.2%) and Black men (68.5%) [2]. Grade 2 and 3 obesity (BMI ≥ 40 kg/m^2^), which is associated with very high risk of adverse health outcomes, is also considerably higher for Black women (27.9%) compared with Black men (14.4%) and non-Hispanic White (16.6%) or Hispanic women (18.9%) [2,3]. Risk of obesity-related illness such as hypertension and diabetes rise markedly as weight status increases [4] and are highest among Black women (35% and 13% respectively) in comparison to all other Americans combined (24% and 9% respectively) [5]. In order to address this pressing public health issue, successful interventions for Black women to prevent weight gain and/or facilitate weight loss are critically needed [6-8].

Most conventional weight loss programs have not been well attended [9], have been less successful with Black and low SES groups [10], and are thought to be less helpful by Black women than Caucasian women [11]. Several clinical trials and observational studies involving a variety of treatment modalities have reported smaller weight losses among Blacks than whites [9,10,12-17]. Trials of lifestyle interventions targeting both white and Black Americans indicate that Black women lose less weight [9,17] and are more likely to regain weight during long-term follow-up [16]. Some of this difference in program effectiveness may relate to participation or adherence variables that could be addressed by cultural adaptation of program design and format [18-20].

Many weight control programs specifically designed for Black Americans have reported at least modest effectiveness [21-30]. Most have used a small group approach that required participants to attend a series of classes at a central location. Such programs may not always be available or affordable or may be poorly attended if class times or locations are not convenient. Alternative approaches adapted to meet the needs of Black women need to be developed or adapted for delivery through channels that are that are inexpensive and more convenient than attending classes at a central location [31,32]. The need may be particularly great for Black women in low income or low SES communities because obesity prevalence is highest at lower income levels and also because the nutrition, physical activity and weight control resources in these communities may be inadequate.

**Figure 1:**
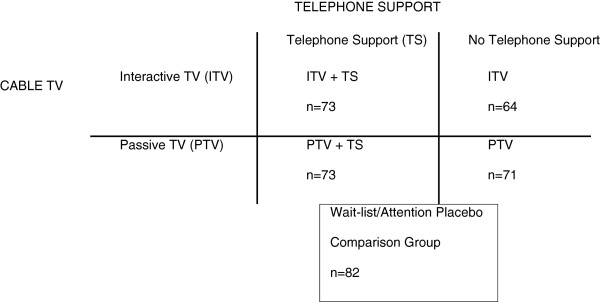
Research design.

Television (TV) is a widely accessible communication channel. The vast majority of Americans own at least one TV, as well as a VCR or DVD player [33-36] and most people [37-44], particularly Black Americans compared with White or Hispanic [45], list TV as a major source of their health information. Routine TV watching can be detrimental to weight control by promoting snacking and sedentary behavior, but can potentially facilitate weight control if effectively used as a “virtual classroom” to deliver a weight control intervention to participants in their home instead of requiring them to travel to a central location. Moreover, television has the ability to demonstrate and model positive behaviors and share stories, images and ideas that reach beyond a traditional classroom setting. This article reports the final outcomes of “Sistertalk, a research project designed to develop and evaluate a culturally-tailored weight control cable TV program for Black women”. The TV program was tested, in randomized fashion, in comparison to a wait-list, attention-placebo comparison condition. The design also allowed for comparison of the effectiveness of program enhancements to a traditional television format in a 2 × 2 factorial comparison of interactive vs. passive TV shows and telephone support or no support. See Figure 1. Versions with or without interactive programming were tested because interactive learning is thought to be more effective than passive learning and has been a prominent theme in culturally adapted programming for low income Black Americans [46]. Versions with and without telephone social support were evaluated because social support is a component of group counseling approaches [47], can provide information, encouragement, emotional support, and enhance environmental supports [48], may facilitate certain aspects of behavior change [49], and may also be necessary for motivation [50]. It was important to see if these add-ons to traditional passive TV programming added value for the additional cost or whether traditional TV without these add-ons could be effective. These were also important questions with respect to future sustainability of the intervention.

## Methods

### Intervention

The SisterTalk study was conducted in Boston, MA. The formative research, design and development of the intervention are discussed in detail elsewhere [51]. Briefly, *SisterTalk* was designed to be culturally appropriate through extensive formative research with over 500 Black women in Boston [51]. The intervention consisted of 12 one-hour weekly programs broadcast live on cable television. Corresponding print materials were mailed out biweekly during the 12-week program. The focus of the program content was not on weight loss per se, but on weight control as defined by each individual woman, which could include weight loss or maintenance of current weight. The intervention was based on Social Action Theory [51,52]. Core content of the TV programs was divided between nutrition and physical activity with behavior change, stress reduction, and self-management principles integrated into both sections [51]. The main goals were to help establish behavior changes in diet and physical activity to improve health (“eat better, move more and feel great”). Thus, the focus was more consistent with a weight control or “health at any size” approach [53,54], than with a traditional weight loss approach [18]. This focus was chosen because of the thoughts and opinions voiced by the target population women during the formative research.

The show was delivered by an all Black female cast including a nutritionist, exercise physiologist and social worker. During the first 40 minutes of each show, the exercise physiologist and nutritionist presented the primary program content, much of which was prerecorded. The diet/nutrition segments of the show focused on decreasing excess calories through decreasing fat and large portion sizes, as well as increasing fruits and vegetables. This included segments demonstrating low fat cooking techniques, shopping, and dining out. The physical activity segments focused on increasing both lifestyle and leisure-time activity; increasing overall movement; and decreasing sedentary behavior. Each show also included a ten minute physical activity break demonstrated with three levels of mobility including high impact, low impact and activities done in a chair. During the last 20 minutes, the social worker led a live “sharing” component, which consisted of a discussion between the social worker, featured guests and live callers. During the discussion, the social worker highlighted the behavioral principles for the week and provided positive reinforcement and modeling of effective problem solving.

### Study design

The evaluation study design (Figure 1) involved a 2 × 2 factorial comparison of interactive vs. passive TV shows and telephone support or no support, with an additional wait list/attention placebo comparison group and random assignment to condition. The experimental conditions were as follows:

#### Interactive TV shows with telephone support (ITV + TS)

Participants in this condition received the 12 weekly TV shows and were given a private toll-free number to call in during the “live” sharing component in the last 15 minutes of the show. They also received 12 weekly then 4 monthly social support phone calls delivered by community outreach educators (COEs). The COEs were Black lay women from the local community in Boston who underwent a 20 hour training process. They called participants several days after each show to check in, discuss progress, provide support and help participants problem solve through any barriers that had arisen during the behavior change process, and to encourage participants to continue moving toward their goals. The COEs were trained to answer simple questions regarding the content, but to ask for help from the research staff if something more complicated arose.

#### Interactive TV shows without telephone support (ITV)

Participants received the same 12-week interactive TV show intervention with the same toll free call-in number, but did not receive the telephone support calls.

#### Passive TV shows with telephone support (PTV + TS)

Participants received the 12 weekly TV shows but their format did not allow them to call in during the sharing segment. They also received 12 weekly and 4 monthly telephone support calls from a COE.

#### Passive TV shows without telephone support (PTV)

Participants received the 12 weekly TV shows, but their format did not allow them to call in during the sharing segment and they did not receive telephone support calls. This condition was similar to regular broadcast TV.

Participants in all four intervention conditions received biweekly mailings of written education materials that corresponded with the TV shows. After the 12 weekly shows, participants in all four intervention conditions received monthly mailings for four months including educational materials and four booster video-tapes that focused on maintenance of behavior changes and included a compilation of the three most popular “10-Minute Workouts” as an exercise video.

#### Wait-list/attention placebo comparison intervention

Participants received biweekly mailings for 12 weeks and then monthly mailings for four months that contained wellness content unrelated to weight, nutrition or physical activity, i.e. cancer screening, injury prevention , etc. They received the entire set of 16 SisterTalk videos and written materials after the 12 month follow-up.

### Participant recruitment and measurement

The study was implemented with four cohorts, or “waves”, of participants. Recruitment methods included flyers and posters distributed at hospitals, clinics, churches, and community retail locations; cable TV public service announcements; and face-to-face and group presentations by staff at churches, housing projects and other community locations. Women were eligible if they self-identified as African American or Black, aged 18–70, resided in the catchment area of the cable TV company, planned to stay in the area for at least one year; were not pregnant or less than four months postpartum, had no physical problems that would prevent mild physical activity; had no previous history of treatment for eating disorders; were able to speak and read English; had no participation in any other weight-control research project; and had access to a working telephone, television and VCR as well as availability to watch the *SisterTalk* cable TV program at its weekly airtime. The study paid for cable TV service for any participants who could not afford it (19% of participants). Women were eligible to participate if they had a BMI ≥ 22.

Prospective participants completed a baseline telephone questionnaire and were then scheduled to attend an in-person screening for additional questionnaires and anthropometric measurements at Brigham and Women’s Hospital or neighborhood health and community centers. Randomization to one took place at the end of the screening and was implemented by asking eligible participants to draw an envelope from a container with concealment of allocation. The targeted sample size was 560, sufficient to permit assessment of a 4 lb (1.8 kg) difference in weight change between any of the experimental groups at 12 months post randomization with 80% power. Participants received a SisterTalk t-shirt and water bottle at baseline.

Follow-up evaluations for all experimental conditions were at approximately 3 months (after the 12-week TV program), 8 months (after the 4-month booster videos) and 12 months. As at baseline, these included a telephone survey followed by an in-person measurement session. Incentives for follow-up completion began at $20 and were later raised to $25 to enhance retention. Moreover, incentives shifted from gifts (cookbooks, fanny packs, etc.) to cash during the study as requested by participants and to increase retention. Participants were followed over the course of the study regardless of whether they participated in all follow-ups in sequence.

### Measures

Height and weight were measured and used to calculate BMI. Survey variables included: demographics, eating habits, and psychosocial measures. The demographic and dietary measures are discussed in detail elsewhere [55,56]. The main dietary outcome measure was the *SisterTalk* Food Habits Questionnaire (STFHQ), which was adapted from older tools [57-61] specifically for this study [55] and was administered by telephone. The STFHQ has excellent test-retest reliability (r = 0.90, p < .001) and validity with fat grams (0.53, p <0.0001) [51,55]. Briefly, the STFHQ consisted of 95 questions including 28 introductory items related to food frequency and 67 fat-related behavioral items about these foods. The “introductory” food questions measured frequency of consumption in the past three months. If a response besides “never” was given, the related behavioral questions were asked (also frequency of consumption). The total FHQ score was calculated as the mean of all behavioral item scores multiplied by the preceding introductory score. Questions that asked about high fat behaviors were reverse scored so that a higher mean score would be reflective of higher fat intake*.*

The Godin Leisure-Time Exercise questionnaire [62], adapted for use in the SisterTalk study was administered during the in-person measurement session. The original Godin scale was modified by specifying 12 activities that were identified as common among Black women during formative research including aerobics, dance, bicycling, walking, jogging, gardening, home exercises, (like jumping jacks or calisthenics), yoga or stretching, housework, weight training, active play with kids, and organized sports. Each participant was asked to report how many days in the previous week the activity was performed (less than once a week to 7 times a week) and the number of minutes performed. Each activity was coded as light, moderate or strenuous. Activities of less than 10 minutes in duration were not included (modified from the original Godin questionnaire which asked about 15 minute bouts of activity). The score was calculated as weekly frequency of that activity multiplied by a factor of 9 (strenuous), 5 (moderate), or 3 (light), and then summed over the 7 days’. The Total Leisure Activity Score (TLAS) was calculated by summing the individual activity scores. TLAS was correlated with accelerometer measurements (r = 0.46, p = 0.0109) in a separate validation study by our team.

### Process evaluation

We monitored the fidelity and dose of intervention delivery. To monitor who was watching an off-camera producer randomly telephoned SisterTalk participants during the show. When one was reached and able to answer a simple question about that day’s content, they won a prize. The producer also recorded the number of ITV group participants who called in during the show. The COEs kept logs of participant telephone support calls that were attempted and completed, and audio-taped calls to participants, which were monitored by a project supervisor on a random basis. In the 3 month follow-up survey, participants were asked what proportion of the shows they had watched and how much of the written materials they had read. In addition, records of requests for mailed videotapes of shows were kept.

### Statistical analyses

Two-way mixed model analysis of variance models were constructed to examine the difference between experimental groups across time points for change (baseline to follow-up) in BMI and primary diet, or physical activity measures. Three-way mixed model analysis of variance models were constructed to examine the interaction between Telephone Support and Interactive/Passive TV treatments over the course of the study. Comparison of intervention groups with the comparison group was accomplished in several different ways and over all time points. First, ITV/PTV groups were compared with the comparison group. Next, TS/No TS groups were compared with the comparison condition. Then individual intervention groups were compared with the comparison group. Finally, all intervention groups were collapsed into a single treatment group and compared to the comparison, using two-way mixed model analysis of variance models to examine the effects of intervention condition versus control across time for each of the primary outcome variables: Weight, BMI, FHQ, and TLAS.

Intent to treat analysis was conducted to estimate group differences after imputing values for participants who were not measured at follow-up. Two sets of models were constructed: the first imputing baseline value for all missing outcomes; the second imputing the sum of the baseline values with the mean change of the Wait-list comparison group. As both methods yielded similar results, the baseline value carried forward method is reported.

## Results

### Study participants

**Figure 2:**
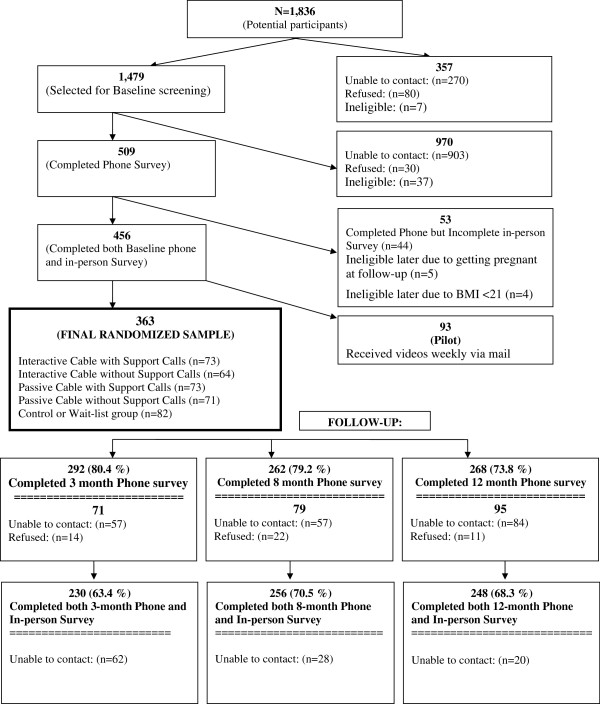
SisterTalk participant recruitment and retention.

A total of 363 women met eligibility criteria, completed both the telephone and in-person measurements and were randomized into the study (Figure 2). Recruitment began three to four months before the baseline telephone survey and a substantial number of women were unable to be re-contacted. Participant characteristics were balanced across the five conditions with no significant differences between groups in any of the demographic characteristics or health conditions (Table 1). The majority of participants (88%) were born in the US, with 84% describing themselves as African American or Black ethnicity, while 9.8% identified as West Indian/Caribbean ethnicity. Most were age 30 or over. Over three quarters were employed either full or part time. The largest group of participants (44%) reported household income from $20,001 and $40,000, though 22% reported $20,000 or less, and 16% reported over $60,000. The majority (59%) lived with children, with 22% of these as without other adults. Forty percent completed a college education or more and only 6% did not complete high school. Over 71% of participants were obese with a fairly even distribution among the classes of obesity. More than one quarter reported having hypertension; 12% reported diabetes and nearly one quarter were on medication for hypertension or a heart condition.

**Table 1:** Demographic profile of randomized participants (percent of each group)

	**Group**					
	**ITV + TS**	**ITV**	**PTV + TS**	**PTV**	**Comparison**	**Total**
	**n = 74**	**n = 66**	**n = 74**	**n = 72**	**n = 82**	**n = 363**
	**%**	**%**	**%**	**%**	**%**	**%**
**Ethnic Group (missing = 25)**						
African	1.5	1.7	1.4	1.6	0	1.2
African Americanblack	86.4	86.7	85.7	73.4	82.1	82.8
American Indian	0	1.6	1.4	3.1	0	1.2
Cape Verdean	3.0	3.3	1.4	4.7	1.3	2.7
Caribbean	6.1	5.0	10.0	12.5	14.1	9.8
Hispanic	1.5	0	0	1.6	0	1.2
Mixed Race/Ethnic Group	1.5	1.7	0	1.6	0	0.9
Other	0	0	0	1.6	0	0.3
**Born**						
In the US	86.3	90.6	90.4	87.3	87.7	88.0
Outside of the US	13.7	9.4	9.6	12.7	12.4	11.6
**Age Group**						
18-29	8.2	15.6	9.6	12.9	11.0	11.3
30-39	27.4	29.7	27.4	25.7	29.3	27.9
40-49	32.9	25.0	31.5	31.4	39.0	32.3
50 or over	31.5	29.7	31.5	30.0	20.7	28.4
**Education**						
Less than High School	6.9	4.7	8.2	4.2	4.9	5.8
High School Graduate	9.6	15.6	12.3	16.9	27.2	16.6
Some College/Technical School	39.7	43.7	31.5	31.2	37.0	37.3
College Graduate	38.8	26.6	34.3	29.6	19.8	27.6
Graduate School	15.1	9.4	13.7	14.1	11.1	12.7
**Household income**						
20,000 or less	23.1	15.3	19.4	26.5	23.0	21.6
20,000-40,000	44.6	47.5	44.8	35.9	47.3	44.1
40,000 – 60,000	18.5	18.6	20.9	18.8	13.5	17.9
60,000 or up	13.9	18.6	14.9	18.8	16.2	16.4
**Employment status**						
Full Time	64.4	73.4	65.3	70.4	65.4	67.6
Part Time	4.1	9.4	11.1	11.3	12.4	9.7
Unemployed	16.4	7.8	6.9	11.3	12.4	11.1
Homemaker/Student	9.6	6.3	8.3	4.2	6.2	6.9
Retired	5.5	3.1	8.32	2.8	3.7	4.7
**Household composition**						
Lives alone	21.9	14.1	19.2	22.5	16.1	18.8
Lives with children only	19.2	15.6	21.9	28.2	24.7	22.1
Lives with adults only	19.2	32.8	19.2	18.3	22.2	22.1
Lives with children and adults	39.7	37.5	39.7	31.0	37.0	37.0
**Weight status (BMI group)**						
Normal (BMI <25)	6.9	7.8	8.2	9.8	11.0	8.8
Overweight (BMI 25–29.9)	20.6	18.8	24.7	18.2	18.3	20.1
Class 1 Obese (BMI 30–34.9)	23.3	21.9	31.5	32.4	29.3	27.8
Class 2 Obese (BMI 35–39.9)	21.9	31.3	19.2	25.4	23.2	24.0
Class 3 Obese (BMI 40 and over)	27.4	20.3	16.4	14.1	18.3	19.3
**Co Morbid conditions** (Doctor ever said you have…)						
Heart Disease	4.2	0	5.6	7.1	1.2	3.6
Heart Attack or Stroke	1.4	0	2.7	1.4	2.5	1.7
Hypertension or High Blood Pressure	26.4	23.4	34.3	28.6	26.3	27.9
Diabetes	9.7	17.2	5.5	18.3	9.9	11.9
Cancer	6.9	3.1	4.1	5.6	2.4	4.4
Medication for Hypertension or Heart Disease	21.9	23.4	26.0	29.0	20.0	24.0
Physical Limitation	26.0	12.5	19.2	18.3	14.6	18.2
**Continuous outcome variables**						
Food Habits Questionnaire (Mean (STD))	1.1 (0.4)	1.0 (0.4)	0.9 (0.4)	1.0 (0.4)	1.0 (0.4)	1.0 (0.4)
Total Leisure Activity Score (Mean (STD))	67.8 (42.5)	67.6 (52.9)	68.7 (48.1)	68.8 (47.9)	60.0 (47.0)	66.9 (47.9)
Body Mass Index (Kg/M^2^) (Mean (STD))	35.6 (8.3)	35.2 (7.5)	34.0 (7.3)	34.1 (7.4)	34.4 (8.5)	34.7 (7.8)
Weight (Kg) (Mean (STD))	97.1 (25.4)	95.5 (20.4)	91.6 (22.6)	91.1 (20.3)	90.9 (22.8)	93.1 (22.5)

Of all enrollees, 63, 70 and 68 were retained for both telephone and in-person surveys at 3, 8 and 12 month follow up respectively, with 80, 77 and 74% completing the telephone survey only, with no statistically significant difference across randomization groups.

### Changes in behavior and BMI

As shown in Table 2, no statistically significant differences were found for interactive versus passive groups or for telephone support versus no telephone support groups in BMI, weight, diet or physical activity changes across time. However, at 3 months each of the four intervention groups showed significantly lower FHQ score compared to the comparison group. At follow-up, these FHQ differences persisted with the ITV still significantly different from the comparison group at 8 months; and PTV + TS still significantly different from the comparison group at 12 months. Higher physical activity was found for the ITV + TS, and ITV groups compared with the comparison group at 3 month evaluation, but not at later evaluations. At 12 months, higher physical activity was found for the PTV group compared with both the comparison group and the ITV + TS groups. At the 3 month follow up, BMI and weight were both significantly lower for the ITV + TS and ITV groups compared with the comparison group.

**Table 2:** Change in major outcome variables from baseline to 3, 8, and 12-month time points for all five experimental conditions at each time point

	**Food habits questionnaire (Fat behaviors)**			**Total leisure activity score (Physical activity)**			**BMI (kg/m**^**2**^**)**			**Weight (kg)**		
Follow-up Month	**3**	**8**	**12**	**3**	**8**	**12**	**3**	**8**	**12**	**3**	**8**	**12**
ITV + TS	−0.29 (0.32)^de^	−0.23 (0.36)^e^	−0.28 (0.33)^e^	11.16 (31.0)^e^	−0.15 (35.9)	−6.58 (42.2)^d^	−0.25 (1.0)^e^	−0.12 (1.2)	0.10 (1.6)	−0.60 (2.6)^e^	−0.31 (3.3)	0.20 (4.4)
ITV	−0.15 (0.31)^e^	−0.17 (0.29)^e^	−0.16 (0.25)	11.89 (64.3)^e^	6.48 (41.5)	0.78 (43.4)	−0.19 (1.1)	−0.39 (1.5)	−0.36 (1.5)	−0.51 (2.9)	−1.08 (3.8)	−1.02 (4.1)
PTV & TS	−0.08 (0.38)^e^	−0.09 (0.36)	−0.19 (0.37)^e^	2.4 (41.7)	−1.8 (43.8)	−1.09 (39.3)	−0.33 (1.2)^e^	0.01 (2.0)	−0.26 (3.8)	−0.88 (3.2)^e^	0.04 (5.3)	−0.71 (10.6)
PTV	−0.12 (0.30)^ae^	−0.17 (0.32)^e^	−0.31 (0.29)^e^	6.15 (37.3)	4.07 (36.4)	10.52 (33.0)^ae^	−0.20 (1.2)	0.03 (1.6)	0.18 (1.5)	−0.60 (3.0)	0.04 (4.2)	0.45 (4.0)
Wait-list Comparison	0.00 (0.34)^abcd^	−0.08 (0.33)^abd^	−0.12 (0.32)^acd^	−3.88 (36.8)^ab^	−0.59 (39.8)	−1.51 (34.3)^d^	0.16 (0.90)^ac^	0.10 (1.36)	0.04 (1.6)	0.46 (2.4)^ac^	0.30 (3.63	0.16 (4.2)
**P-value** (Group difference)	<.0001	.0306	.0121	.2255	.6730	.0594	.1416	.4951	.7247	.1083	.4478	.7457

Footnotes: *ITV* interactive television, *PTV* passive television, *TS* telephone support.

p < .05 different from group as follows: ^a^different from ITV + TS, ^b^different from ITV, ^c^different from PTV + TS, ^d^different from PTV, ^e^Comparison.

All data show include imputation for missing follow-up data using the baseline carried forward method.

With the four active interventions combined, i.e., comparing participants with access to the TV intervention in any format with those in the comparison group (Table 3), the TV intervention group was associated with statistically significant decreases in BMI and dietary fat and increased physical activity at 3 months. For dietary fat, but not BMI or physical activity, the differences remained significant at 8 and 12 month follow-up.

**Table 3:** Change in major outcome variables from baseline to 3, 8, and 12-month time points for comparison versus collapsed intervention groups

	**Food habits questionnaire (Fat behaviors)**			**Total leisure activity score (Physical activity)**			**BMI (kg/m**^**2**^**)**			**Weight (kg)**		
**Follow-up Month**	**3**	**8**	**12**	**3**	**8**	**12**	**3**	**8**	**12**	**3**	**8**	**12**
**Comparison Group** (Mean, Std dev)	0.00 (0.28)	−0.06 (0.28)	−0.09 (0.28)	−2.75 (30.9)	−0.40 (32.8)	−1.07 (28.8)	0.11 (0.77)	0.07 (1.12)	0.03 (1.33)	0.32 (2.03)	0.20 (3.00)	0.12 (3.55)
**Intervention Group** (Mean, Std dev)	−0.10 (0.28)	−0.12 (0.30)	−0.17 (0.28)	4.62 (34.8)	1.47 (33.2)	0.49 (32.7)	−0.15 (0.87)	−0.01 (1.33)	−0.06 (1.94)	−0.40 (2.3)	−0.24 (3.53)	−0.18 (5.40)
**P-value** (Group difference)	<.0001	.0053	.0034	.0335	.4084	.3950	.0134	.3292	.7260	.0085	.2788	.7240

All data show include imputation for missing follow-up data using the baseline carried forward method.

### Process evaluation

The data in Table 4 provide insights about intervention dose for participants in the TV intervention groups. In the four month follow-up survey, over two thirds (69.3%) reported having watched 7–12 shows of the initial 12 show series. Groups receiving telephone support were more likely to report watching higher numbers of shows (p = .0297) (data not shown). Watching a higher number of shows was associated with lower dietary fat at 3 months (FHQ score) (p = .0105), which persisted to the 12 month follow-up (p = .0076). Number of shows watched was not associated with differences in physical activity score or BMI at either 3 or 12 month follow-up.

**Table 4:** Intervention dose: intervention participant viewing of TV shows and reading of written materials

**Shows watched**		
	n = 166	%
None	5	3.0
1-3 shows	6	3.6
4-6 shows	40	24.1
7-9 shows	50	29.5
10-12 shows	66	39.8
**Written materials read**		
	n = 166	%
None	0	0
A Few	30	18.0
Some	41	24.7
Most	48	28.9
All	47	28.3

Over half (57%) reported having read most or all of the written materials. The PTV group reported having read fewer materials than the other three groups, but the difference across groups was not statistically significant (p < .09, data not shown). Overall, reading more written materials was associated with lower dietary fat (FHQ score) (p = .0054) at 3 months, which did not persist to the 12 month follow-up (p = .1876). As with viewing of the cable TV show, reading was not associated with differences in physical activity or BMI at any of the three follow-up time points.

Neither of the experimental manipulations was fully implemented as intended. In the interactive conditions, women were reluctant to talk “on-air”. Only a few women actually called-in to ask questions, so instead program producers called participants to solicit questions. But overall, the amount of interaction was limited. For the telephone support component, a total of 12 different COEs were employed over the course of the project. Some participants had the same COE for the entire intervention; others had as many as three different COEs calling them. Missing data limited the ability to assess how many of the possible 2336 calls (146 participants × 16 expected calls) were actually completed. No process evaluation forms were completed for 590 (25%) calls. COEs were given tape recorders to record calls, but tapes were often missing for calls (e.g. COEs said they had forgotten to turn the device on or that it had not worked). Even when calls were attempted, participants were often not home or unable to talk because life issues often took precedence over participating in weekly support calls.

For the 48.2% of participants with call data, less than half of the 16 calls were completed. For these participants, the number of attempts per call (whether completed or not) was 2.33, and the average length of completed calls was 17.1 minutes. Direct supervision of COEs was difficult due to the geographical distance between the COEs (Boston) and project management staff (Rhode Island).

## Discussion

The SisterTalk study was designed to evaluate the effectiveness of a culturally tailored weight control program delivered by cable TV and also to test the separate and combined effects of two potential strategies (interactive programming and provision of telephone support) for enhancing effects. The overall results indicate modest effects of the TV interventions, taken together, in lowering BMI and dietary fat intake and increasing physical activity relative to no intervention when assessed soon after delivery of the 12 week program, but only the effect on dietary fat intake was sustained for the remainder of the year. The cultural tailoring and inclusion of theoretical constructs seems to be effective, but cannot be compared to a non-tailored intervention, or one tailored on theoretical constructs with this design. Kreuter, et al. found that cultural tailoring combined with tailoring on theory-based psychosocial constructs was more effective than tailoring on theory-based constructs alone [63], but further research should replicate these findings.

The finding of significant initial effects in the expected direction and for both behavior changes and BMI is encouraging as to the potential utility of this general approach with Black women. Effects on weight were modest (0.65 kg weight loss at 3 months. This is likely attributable mostly to the program content, which allowed flexible goal setting and emphasized weight control more strongly than weight loss, as described under Methods. Process evaluation data indicating that two-thirds of the women (70%) actually watched most of the shows or read most of the written materials (57%), which may also explain the modest changes observed, however group attendance in other programs is often at 70% or lower [21,25,64,65]. An effect of intervention dose was evident for dietary fat intake, and dietary fat intake was also the outcome variable showing the most responsiveness to the intervention overall, over time, and to the intervention enhancements. While differences in fat intake persisted over time, this did not translate into a sustained difference in weight or BMI. Thus, future enhancements of the dietary content could include a stronger focus on reducing overall caloric intake and portion control, which may have a stronger effect on weight than reducing dietary fat intake.

The longer term findings clearly indicate a need to strengthen intervention delivery after the initial period in order to achieved sustained effects on BMI. Several studies that have included Black women report that initial weight loss is a major predictor of longer term weight loss [66,67], which suggests that alterations to improve the potential for early weight loss may also improve results over the longer term. This could involve identifying culturally salient strategies to motivate overweight and obese women to lose weight rather than only control weight initially and strengthening advice regarding how to do this.

The attenuation of physical activity effects over time suggests that strengthening the physical activity intervention may be particularly important. Physical activity is difficult to measure [68,69], and studies of the validity of PA assessments in Black populations are limited [70], but it does not seem likely that measurement issues would explain attenuation of effects over time and the attenuation over time is consistent with the similar finding for BMI. National survey data indicate that Black women report lower levels of leisure time PA as well as higher levels of sedentary behavior compared to white women [71,72]. Physical activity has been identified as an important predictor of weight maintenance overall [73,74], and in observational data for Black women [75], although physical activity levels did not predict weight maintenance among Black women in a controlled trial [76]. In any case, it is likely that the intervention failed to achieve enough change during the initial program period to be sustained over time. The main activity message was to increase activity during daily life, but this message did not seem specific enough, and the exercise portion on the TV show was only 10 minutes. Future interventions should advocate more specific activity increases that can be planned and carried out consistently.

Other ways to improve behavior change and/or enhance maintenance of changes might include more gradual tapering of program frequency, e.g. providing additional shows bi-weekly before reducing the frequency to monthly, repackaging and re-airing core content, or combining these strategies. Another possibility would be developing a sufficient number and variety of shows to permit ongoing weekly access. Given the advent and popularity of digital communication channels in recent years, producing shows with web-based delivery might be another strategy. These approaches would be compatible with an entirely home-delivered program. The exercise component of the shows could also be given greater emphasis, e.g., by adding a set of exercise tapes rather than only including an exercise component in a multifocal TV show and encouraging more walking throughout the day.

Adding interactive programming or telephone support did not appear to improve effectiveness. The one exception was for fat intake, which improved among all of the experimental groups at most time points. The absence of any measurable effects of the interactive and social support components was disappointing given that each of these had a sound theoretical basis. For both components we can attribute the lack of effects at least, in part, to incomplete implementation, as indicated by the process evaluation. Omitting the interactive component would be favorable from feasibility, replicability, and cost perspectives in that future programming could be delivered via prerecorded rather than live programs. However, the aforementioned considerations related to how to strengthen the intervention would still need to be addressed. Perhaps interactivity could be added with a telephone hotline, an internet chat-room or email/texting. With respect to the telephone support, the feasibility issues encountered were not entirely unexpected. Non-completed calls have been an issue with telephone counseling in other research, [77-79] but it is difficult to disentangle issues of access to participants, from passive refusal to participate in that portion of the intervention. Updated research is needed in this respect given that the current context for telephone communications has changed dramatically: fewer people have telephone land lines, cell phones may be answered at times when people are not at home, and caller identification makes it easier to be selective about which calls are answered [80]. Also, as noted above, the promise of digital strategies for remote support interventions such as internet chat rooms might be relevant [81,82]. On the other hand, approaches that involve some direct personal contact or networking among participants would also be worth evaluating. An original plan for networking of participants for “out of class” get-togethers proved infeasible based on geographic dispersion of the sample and concerns about contamination of the waiting list comparison group and subsequent cohort waves. However, inability to join with a friend or network with others in the study were the most frequent criticisms of the program by participants, as women wanted to work with others as part of the program. Thus, it might be helpful to add a social component to future interventions.

The experience the COEs was mixed, raising questions that require careful consideration about whether or how to incorporate peer-counselors in this type of intervention. Involvement of the COEs was invaluable during the formative assessment phase of the project, e.g., provider “insider” perspectives of many aspects of the intervention and facilitating recruitment through personal connections in the targeted neighborhoods. However, their performance was less predictable when the role shifted to conducting peer counseling that involved specific skills, meeting deadlines, and recording and reporting data. Lessons learned include the need to have different criteria for hiring, training, and supervising outreach workers hired for formative activities and recruitment vs. counseling. Other researchers have had success with lay counselors when strict screening, training and certification procedures were used [77-79,83-89]. Consistent with this interpretation, several COEs resigned as poor performance was brought to their attention and more was demanded from them. Thus, when forming partnerships with representatives of a targeted community, a great deal of care is necessary to match the skills and experience of the community members employed with the skills and abilities required for the specific tasks they are going to be responsible for.

Strengths of this study include the randomized design with multiple follow-ups; the extensive community-engaged formative research used to plan all aspects of the study; the comprehensive cultural-tailoring of the intervention; and the use of valid culturally adapted evaluation measures. Moreover, the targeting of Black women is a strength given the marked disparities in BMI and health outcomes.

Limitations include the aforementioned incomplete delivery of the intended intervention dose and our inability to completely judge the quality of telephone support. In addition, the lower than planned recruitment is a limitation given that it reduced the statistical power to see significant effects on outcomes, particularly for comparisons between the different intervention formats. However, as discussed earlier, because the intervention developed with target audience input ultimately focused on weight control rather than weight loss, a higher sample size would likely not have mattered with respect to the weight outcome; however, more power would likely have enhanced the significance of the other observed changes. Future weight control intervention studies need to find ways to improve recruitment of Black women.

Yancey, et al., found that Black women of higher weight status were more likely to be recruited by face-to-face engagement [90,91]. While we conducted some face-to face recruitment, this was somewhat limited by the geographic distance of the research team and relied mostly upon the COEs for recruitment. A bias toward recruitment of higher SES women is implied by the levels of education, employment, and income, although higher SES African Americans do tend to live in lower income neighborhoods than might be expected [92]. Having a higher SES sample would not affect internal validity in our randomized design, but would potentially affect salience of the intervention content, which was informed by formative research with a lower income sample. This lack of correspondence between the audience the intervention was developed for and the sample that actually joined the study, could also have limited effectiveness. Attrition was also moderate (>30% for in-person follow-up measurements). While the follow-up incentives were raised and shifted from gifts to cash over-time and this improved participation retention, overall retention was still not ideal. More research is needed on how to retain participants, especially Black women in weight control studies.

## Conclusions

In summary, this cable-TV delivered intervention was successful in improving participants’ eating habits in the short and long term as well as improving physical activity and modestly decreasing weight in the short term. The SisterTalk cable TV intervention achieved weight maintenance or modest weight loss, which was the objective of the intervention, but sustained weight loss did not occur. We also found that interactive/live TV and telephone support from lay educators also enhanced the video intervention with respect to dietary fat outcomes, but were not associated with any physical activity or weight related effects. The smaller differences in physical activity and sustained changes in weight and BMI may have been achieved better with stronger participant recruitment and retention as well as enhancing the dose of the intervention received by participants. Future research should build on the successes and lessons learned by enhancing viewership of the shows and use of the materials while maintaining and strengthening the content related to calorie reduction and physical activity, using video or web-based streaming video rather than a live TV format to allow for more flexible and repeat viewing, extending the length of the intervention, and finding ways to allow for networking of study participants.

## Abbreviations

BMI: Body mass index; ANOVA: Analysis of variance; SES: Socioeconomic status; TV: Television; ITV + TS: Interactive TV shows with telephone support; ITV: Interactive TV shows without telephone support; PTV + TS: Passive TV shows with telephone support; PTV: Passive TV shows without telephone support; COEs: Community outreach educators; STFHQ: SisterTalk food habits questionnaire; TLAS: Total leisure activity score.

## Competing interests

The authors declare that they have no competing interests.

## Authors’ contributions

KG and PMR made substantial contributions to the planning and implementation of the SisterTalk intervention and overall study, acquisition and interpretation of data, and preparation of all sections of the manuscript. TL was the principal investigator of the study and contributed to the discussion section of the paper prior to his death. SK was Co-Principal Investigator of the study and provided guidance throughout all aspects of the project planning and implementation. She also made substantial contributions to the paper especially the introduction and discussion sections. UK and SA both made significant contributions to the analysis and interpretation of data. All authors read and approved the final manuscript.
